# Supplementary material for: Active carpets drive non-equilibrium diffusion and enhanced molecular fluxes
Source: Nat Commun. 2021 Mar 26;12:1906. doi: 10.1038/s41467-021-22029-y (PMC7997990; doi:10.1038/s41467-021-22029-y)
Supplement: Supplementary file 2 — Description of Additional Supplementary Files [file 41467_2021_22029_MOESM2_ESM.pdf]

Description of Supplementary Files

Active carpets drive non-equilibrium diffusion and enhanced molecular fluxes

File Name: Supplementary Movie 1

Active fluctuations generated by a carpet made of perpendicular Stokeslets. A large number of actuators on a surface (e.g. filter-feeding organisms) generate flows, pulling the liquid down (green arrows) or pushing it up (red arrows), with dynamics according to independent Ornstein Uhlenbeck processes. The total flow $\boldsymbol{v}$ generated by all these actuators is shown by a blue arrow above the carpet. The ellipsoid represents the strength of these fluctuations, with horizontal semi-minor axes equal to $\sqrt{\langle v_x^2 \rangle} = \sqrt{\langle v_y^2 \rangle}$ and a vertical semi-major axis equal to $\sqrt{\langle v_z^2 \rangle}$. Simulations are performed as described in Methods §4.

File Name: Supplementary Movie 2

Sedimentation of particles towards an active carpet. An ensemble of particles with sedimentation velocity $v_{\mathrm{g}} = 1$ are released near an active carpet (black surface). Far from the carpet the particles fall under gravity, but nearby they are repelled by active fluctuations. This effect gives rise to a sedimentation profile that does not follow the Boltzmann distribution. The particles do not interact with each other, and they all experience independent fluctuations. Simulations are performed as described in Methods §8.

File Name: Supplementary Movie 3

Diffusion from a source towards an active carpet sink. Particles are released from a source (top black surface), after which they are subject to active fluctuations, without sedimentation, $v_{\mathrm{g}} = 0$. When a particle hits the sink (bottom black surface) it is placed back at the source. The number of crossings is marked with the following colours: orange (0), blue (1), yellow (2), green (3), magenta (4), cyan (5), and red (6). The particles do not interact with each other. Simulations are performed as described in Methods §11.
